# Direct carotid cavernous sinus fistulae: vessel reconstruction using flow-diverting implants

**DOI:** 10.1007/s00062-016-0511-6

**Published:** 2016-04-29

**Authors:** C. M. Wendl, H. Henkes, R. Martinez Moreno, O. Ganslandt, H. Bäzner, M. Aguilar Pérez

**Affiliations:** 1 Neuroradiologische Klinik, Neurozentrum, Klinikum Stuttgart, Stuttgart, Germany; 2 Institut für Röntgendiagnostik, Zentrum für Neuroradiologie, Universitätsklinikum Regensburg, Regensburg, Germany; 3 Neurochirurgische Klinik, Neurozentrum, Klinikum Stuttgart, Stuttgart, Germany; 4 Neurologische Klinik, Neurozentrum, Klinikum Stuttgart, Stuttgart, Germany; 5 Medizinische Fakultät, Universität Duisburg-Essen, Essen, Germany

**Keywords:** CCF, Endovascular, Flow diverter, Pipeline, p64

## Abstract

**Purpose:**

Retrospective evaluation of our experience with the use of flow diverters (FD) for the endovascular treatment of direct carotid-cavernous sinus fistulae (diCCF).

**Methods:**

Between 2011 and 2015, 14 consecutive patients with 14 diCCF were treated with FD alone or in combination with other implants in a single institution.

**Results:**

A total of 21 sessions were performed in 14 patients. FD placement was technically successful in all cases without an adverse event. Patients were treated with FD alone (*n* = 5), FD and covered stents (*n* = 2), FD and coils (*n* = 7). A total of 59 FD (24 Pipeline Embolization Device, Medtronic; 35 p64 Flow Modulation Device, phenox), 291 coils, and 3 stent grafts were used. Three of 14 diCCF were completely occluded after the 1^st^ session, a minor residual shunt was found in 7/14, and in the remaining 4/14 patients, the shunt volume was reduced significantly. The mean follow-up period encompassed 20 months. Additional treatment included transvenous coil occlusion (*n* = 3) and/or further FD deployment (*n* = 5). An asymptomatic internal carotid artery (ICA) occlusion was encountered in 2 patients, related to an interruption of antiaggregation. At the last follow-up, 10/14 patients were free from ocular symptoms (71 %), 2 had residual exophthalmos, and no patient had clinical deterioration.

**Conclusion:**

The usage of FD for the treatment of diCCF is straightforward. Injury of the cranial nerves can be avoided. In most cases, ocular symptoms improve. Several FD layers and/or an adjunctive venous coil occlusion are required. Complete occlusion of a diCCF may take weeks or months and long-term antiaggregation is required. In the future, a flexible stent graft might be a better solution.

## Introduction

Carotid-cavernous sinus fistulae (CCF) in general represent an abnormal communication between the internal and/or external carotid arteries and the cavernous sinus, resulting in venous congestion of the cavernous sinus and the adjacent veins and sinuses. Clinical presentation may include chemosis, conjunctival injection, visual impairment, and diplopia. According to the Barrow classification, CCFs are subdivided into four different types (A–D), depending on flow rates, etiology, and source of feeding vessels. Direct carotid-cavernous sinus fistulae (diCCF, type A) are generally characterized by an abnormal arteriovenous communication between one internal carotid artery (ICA) and the ipsilateral cavernous sinus [1]. diCCF are typically due to a severe blunt or penetrating head trauma [2]. Less frequently they occur spontaneously in the context of vessel wall connective tissue disease (e. g., Ehlers Danlos syndrome) or after the rupture of a preexisting cavernous ICA aneurysm [3–5]. Moreover, diCCF can be a complication following skull base surgery or endovascular procedures adjacent to the cavernous segment of the ICA (e. g., balloon angioplasty or stent placement) [6–10].

The goal of the treatment of diCCF is to occlude the arteriovenous shunt and to preserve the patency of the concerned internal carotid artery (ICA). A variety of endovascular treatment strategies have been developed during the past three decades, geared to the size and location of the fistulous point, as well as to the venous outflow patterns. According to the classification of van Rooij et al. [5], diCCF can further be classified with regard to the shunt volume in high-, intermediate-, or low-flow fistulae. In high-flow diCCF, the entire shunt volume enters the arteriovenous fistula without filling of intracranial vessels. In intermediate-flow diCCF, both the diCCF and the ipsilateral intracranial vessels are supplied with blood from the ICA. In low-flow diCCF, only mild arteriovenous shunting to the cavernous sinus exists. Historically, endovascular treatment strategies via transvenous or transarterial access routes included the use of detachable balloons [4, 11], coils [12–14], covered stents [15, 16], and liquid embolic agents [17, 18]. Silicon balloons were widely used for the treatment of diCCF over many years, until their unexpected withdrawal from the market in 2004. Today, detachable coils are most frequently used. In the case of a large shunt volume, an adjunctive device such as a covered stent may be considered.

Initially, flow diverting implants were designed for the extrasaccular treatment of complex cerebral aneurysms, but techniques and indications for the use of FD continue to evolve [19]. Recently published case reports showed promising results for the treatment of diCCF with flow diverter (FD) [20–22]. The dense coverage of FD not only provides protection of the ICA during transvenous coil insertion, but also enables endothelial overgrowth of the lacerated arterial segment.

We sought to retrospectively evaluate our experiences with the use of FD for endovascular treatment of diCCF. Beside some sporadic case reports, this is, to the best of our knowledge, the first case series reporting the use of FD for the treatment of diCCF.

## Materials and methods

### Patient enrollment and adherence to ethical standards

Being intrigued by the capacity of FD to reconstruct diseased arteries, we offered this treatment to all elective patients with diCCF who had been referred to or diagnosed by us since April 2011. In patients with an iatrogenic diCCF sustained during an endovascular procedure (e. g., stent angioplasty), the FD reconstruction of the concerned artery was part of the bailout strategy. All patients and/or their legal representatives were informed about the disease and the treatment options, and declared consent in written form. In retrospect, we analyzed all concerned case histories and endovascular procedures until June 2015. The responsible ethics committee (“Ethik-Kommission bei der Landesärztekammer Baden-Württemberg”) issued a waiver for ethical consultation for this retrospective data analysis. For diCCF following the rupture of a cavernous ICA aneurysm into the cavernous sinus, all currently available FD can be used on-label. In diCCF due to an endovascular or external trauma, only p64 Flow Modulation Device (phenox, Germany) can be applied on-label. The approved usage of Pipeline Embolization Device (PED, Medtronic, Ireland), flow redirection endoluminal device (FRED, Microvention, USA), and Surpass Streamline Flow Diverter (Stryker, USA) is limited to the treatment of aneurysms.

### Diagnosis of diCCF

Work-up of patients referred to us or identified as carrying a diCCF due to typical signs and symptoms (e. g., chemosis, exophthalmos, glaucoma, and/or progressive visual loss) underwent CT and/or MRI/magnetic resonance angiography (MRA) examinations of the head, according to clinical standards. Digital subtraction angiography (DSA) including runs with increased frame rate and manual cross compression was used to confirm the diagnosis, identify the site of the ICA tear, and understand the patterns of venous drainage. If a diCCF occurred as a complication of an intracranial endovascular procedure, no further imaging besides DSA was performed in order to not delay the necessary treatment. Finally, all diCCF were classified into high-, intermediate- and low-flow fistulae according to the grading scale of van Rooij et al. [5].

### Endovascular treatment

The technical details of the procedures followed generally accepted principles, based on the decisions of the operator and/or the senior author (HH). diCCF presenting with a small shunt volume were initially treated with FD alone. In diCCF where the fistulous point could be identified clearly and where the ICA was not too tortuous, the deployment of a stent graft was attempted in order to occlude the fistulous point immediately. In fistulae with an intermediate or large shunt volume, FD deployment was combined with either transarterial or transvenous coil occlusion of the cavernous sinus and the efferent veins.

Prior to FD placement, clopidogrel (single 600 mg loading dose, thereafter 75 mg/d) and aspirin (single 500 mg loading dose, thereafter 100 mg/d) were administered for dual platelet antiaggregation. The level of platelet function inhibition was assessed using the Multiplate Analyzer (Roche Diagnostics, Switzerland). In case of insufficient response to clopidogrel, a second loading dose of 180 mg ticagrelor (Brilique; AstraZeneca, United Kingdom) was given per os (PO), followed by 2 × 90 mg ticagrelor daily thereafter. If the diCCF occurred as a complication during an endovascular procedure in a patient not already being loaded or premedicated, an intravenous bolus injection of 500 mg aspirin and 180 mg ticagrelor via a gastric tube were given, combined with a body weight-adapted intravenous bolus injection of eptifibatide (Integrilin; GlaxoSmithKline, United Kingdom). A carotid occlusion test was performed before treatment in order to visualize the collaterals for the concerned ICA in the event that the sacrifice of this vessel would become necessary. Under general anesthesia and with full relaxation, a 6F guiding catheter was placed in the cervical segment of the concerned ICA. Then a 0.027” ID microcatheter (e. g., Marksman, Reverse 27, both Medtronic; Excelsior XT27, Stryker) was navigated over a 0.014” microguidewire (e. g., Synchro2 0.014”, Stryker) across the fistulous point to the M1 segment of the ipsilateral middle cerebral artery. For coil occlusion a second microcatheter (e. g., Excelsior SL10, Stryker; Echelon10, Medtronic) was placed in the cavernous sinus from either the arterial or venous side, and coil occlusion was performed before (transarterial) or after (transvenous) the FD were deployed. The number of FD used in each patient depended on the periprocedural evolution of the shunt reduction, based on the judgement of the operator. When the shunt volume had been significantly reduced, the endovascular treatment was terminated.

### Follow-up and outcome

Angiographic and clinical follow-up assessment was scheduled 3, 6 and 12 months after the treatment, and annually thereafter. The clinical examination was carried out by a board-certified neurologist or neurosurgeon. Additionally, patients were advised to immediately return to our institution in case of any clinical deterioration. The presented data were collected retrospectively from inpatient hospital records, radiologic reports, and DSA examinations. Angiographic outcomes were categorized as “target vessel occlusion”, “complete diCCF occlusion”, or “incomplete diCCF occlusion with persistent flow of the diCCF”. Clinical outcome was reported for every follow-up and graded into four types: 1) full recovery from neurological symptoms, 2) improved neurological symptoms, 3) unchanged neurological symptoms, or 4) worsening of symptoms.

## Results

### Patients

Between April 2011 and June 2015, a total of 14 patients (3 men, 11 women; median age 59 years, age range 18–84 years) with 14 diCCF were treated with FD alone, or in combination with coils or covered stents at a single institution. In 2 patients the diCCF occurred spontaneously and 4 patients had a trauma prior to the onset of symptoms. One patient presented with a spontaneous rupture of a cavernous ICA aneurysm, which resulted in a diCCF. In 7 patients the diCCF occurred as a complication of an endovascular treatment (Tab. 1 and 2).

**Tab. 1:** Etiology of direct carotid-cavernous sinus fistulae and shunt volume

Patient #	Etiology	Shunt volume
1	During stenting	High
2	During stenting	Intermediate
3	During stenting	Low
4	Trauma	Intermediate
5	During stenting	Low
6	Spontaneous	Intermediate
7	Trauma	Intermediate
8	During stenting	Low
9	Spontaneous	High
10	Trauma	Low
11	Trauma	High
12	During stenting	Low
13	Aneurysm rupture	Intermediate
14	During stenting	Low

**Tab. 2:** Initial treatment, follow-up results and retreatments in 14 patients with direct carotid-cavernous sinus fistulae

Patient Number of treatments	*Etiology of diCCF* Initial treatment	Follow-up results	Retreatment Clinical outcome
#1 male, 74 years 1 procedure	*Stent PTA* 2 × SG (Graftmaster Coronary Stent Graft [Abbott Vascular, USA] 1 × 4/12, 1 × 4/16) 4 × FD (PED: 3 × 3.75/20, 1 × 4/20)	6 d: incomplete diCCF occlusion 207 d: complete diCCF occlusion 456 d: complete diCCF occlusion	None; 456 d: ICA occlusion due to interruption of medication intake mRS 0
#2 female, 76 years 2 procedures	*Stent PTA* 2 × FD (1 × PED 3/2, 1 × p64 3.5/15)	4 d: incomplete diCCF occlusion 14 d: further diCCF occlusion 161 d: complete diCCF occlusion 1210 d: complete diCCF occlusion	One; 70 d: residual AV shunt, transvenous coil occlusion (12 coils) mRS 0
#3 male, 72 years 1 procedure	*Stent PTA* 1 FD (1 × p64 3.5/18)	134 d: complete diCCF occlusion, ISS 246 d: complete diCCF occlusion, ISS 328 d: complete diCCF occlusion, ISS 545 d: complete diCCF occlusion, ISS	One; 136 d: re-stent PTA mRS 3 after ICA TEA, unrelated to the diCCF
#4 female, 17 years 2 procedures	*Severe head trauma* 24 fibered coils, transarterial 6 × FD (5 × p64 4.5/18, 4/21, 4/18, 4/18, 4.5/15, 1 × PED 3.5/14)	5 d: incomplete diCCF occlusion 9 d: incomplete diCCF occlusion 65 d: complete diCCF occlusion 252 d: complete diCCF occlusion	One; 5 d: 4 × FD (2 × PED 3/20, 2 × p64 3/15) mRS 0
#5 male, 60 years 1 procedure	*Stent PTA* 2 × FD (2 × p64 4/18)	44 d: complete diCCF occlusion 381 d: complete diCCF occlusion, ISS 457 d: complete diCCF occlusion, ISS 503 d: complete diCCF occlusion, ISS	One; 381 d: re-PTA mRS 0
#6 female, 50 years 2 procedures	*Severe head trauma* 3 × FD (1 × p64: 2 × 4,5/24, 1 × p64 4/24)	7 d: incomplete diCCF occlusion 18 d: incomplete diCCF occlusion 190 d: complete diCCF occlusion 545 d: complete diCCF occlusion	One; 20 d: 3 × FD (1 × PED 3,25/16, 2 × p64 3,5/18) mRS 0
#7 female, 66 years 2 procedures	*Severe head trauma* 2 × FD (2 × PED 5/20) 65 coils, transvenous	39 d: incomplete diCCF occlusion 63 d: incomplete diCCF occlusion 125 d: complete diCCF occlusion 957 d: complete diCCF occlusion	One; 63 d: 1 × FD (1 × p64 4/18 mm); 9 coils, transvenous mRS 1
#8 female, 44 years 1 procedure	*Stent PTA* 1 × SG (Graftmaster 1 × 3.5/12) 2 × FD (PED 1 × 4/20, 1 × 3.5/18)	7 d: incomplete diCCF occlusion 96 d: complete diCCF occlusion 924 d: complete diCCF occlusion	None mRS 2, due do previous cerebral ischemia from bilateral chronic ICA occlusion
#9 female, 74 years 3 procedures	*Spontaneous* 65 fibered coils, transvenous	2 d: incomplete diCCF occlusion 26 d: incomplete diCCF occlusion 76 d: incomplete diCCF occlusion 521 d: complete diCCF occlusion	Two: 2 d: 2^nd^ session: 8 FD (8 × PED: 2 × 5/25, 3 × 4.75/20, 1 × 4.5/35, 2 × 5/30); 26 d: 3^rd^ session: 20 fibered coils, transvenous mRS 0
#10 female, 64 years 1 procedure	*Severe head trauma* 3 fibered coils, transarterial 1 × FD (p64 1 × 4.5/21)	77 d: complete diCCF occlusion 658 d: complete diCCF occlusion	None mRS 0
#11 female, 54 years 1 procedure	*Severe head trauma* 85 coils, transarterial 6 × FD (p64: 1 × 4/21, 1 × 3,5/18, 1 × 3,5/15, 1 × 4/15, 2 × 3.5/21)	2 d: incomplete diCCF occlusion 12 d: incomplete diCCF occlusion 18 d: ICA occlusion	None; 18 d: ICA occlusion due to interruption of medication intake mRS 1, due to the head trauma
#12 female, 58 years 1 procedure	*Stent PTA* 1 × FD (PED 1 × 4/20)	73 d: complete diCCF occlusion 737 d: complete diCCF occlusion	None mRS 0
#13 female, 86 years 1 procedure	*Ruptured cavernous ICA aneurysm* 8 × FD (6 × p64: 2 × 4.5/21, 1 × 4.5/18, 1 × 4.5/15, 1 × 4/18; 2 × PED: 1 × 4.5/25, 1 × 4/20) 35 coils, transvenous	142 d: complete diCCF occlusion	None mRS 1
#14 female, 46 years 2 procedures	*Stent PTA* 3 × FD (3 × p64 2 × 3.5/18, 1 × 4/18)	3 d: incomplete diCCF occlusion 8 d: incomplete diCCF occlusion 382 d: complete diCCF occlusion	One; 6 d: 2 × FD (2 × p64 1 × 3.5/18, 1 × 4/18) mRS 0

All sizes are given in mm; all time intervals refer to the first treatment

*diCCF* direct carotid-cavernous sinus fistula, FD flow diverter, *ICA* internal carotid artery, *ISS* in-stent stenosis, *mRS* modified Rankin Scale, PED Pipeline Embolization Device (Medtronic, Ireland), *p64* Flow Modulation Device (phenox, Germany), *PTA* percutaneous transluminal angioplasty, *SG* Stent Graft

### Primary treatment results

**Fig. 1:**
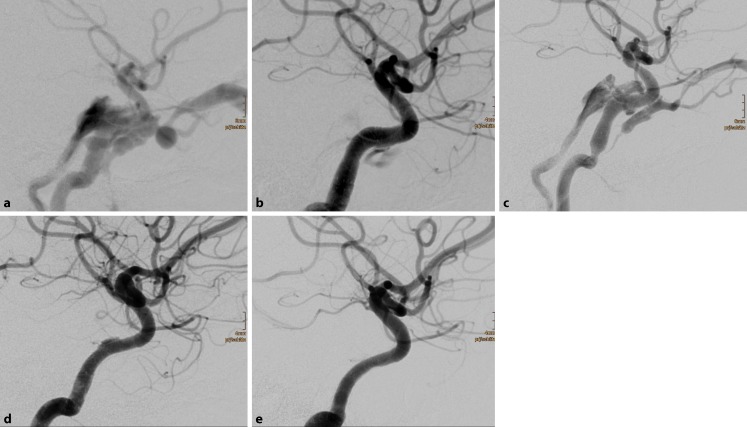
Spontaneous direct carotid-cavernous sinus fistula in a 50 year-old female patient (**a**). Deployment of three flow diverters resulted in a significant reduction of the shunt volume (**b**). Significant recurrence of the arteriovenous shunt prompted a second treatment 19 d later, with implantation of another three flow diverters (**c**), without an instantaneous angiographic effect. Due to a significant clinical improvement, the patient decided to return home. Follow-up digital subtraction angiography examinations 6 months (**d**) and 18 months (**e**) later confirmed the occlusion of the arteriovenous fistula

**Fig. 2:**
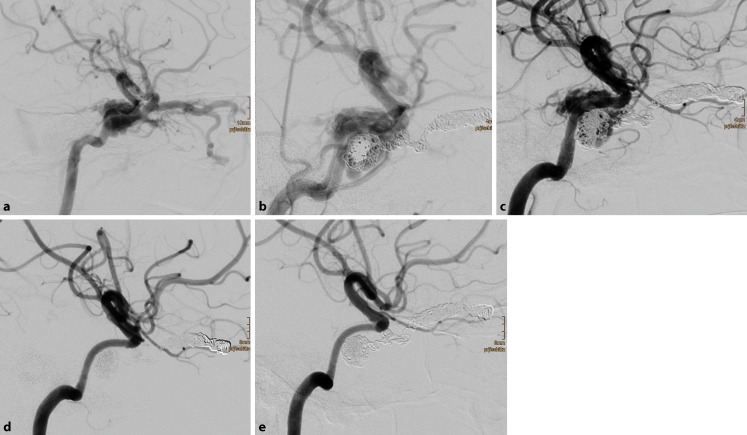
Traumatic direct carotid-cavernous sinus fistula in a 17 year-old female patient after severe head trauma (**a**). Chemosis, exophthalmos, and elevated intraocular pressure were due to massive arteriovenous shunt from the right internal carotid artery (ICA) into the adjacent cavernous sinus. Endovascular treatment in one session comprised insertion of 24 detachable coils with nylon fibers into the right superior ophthalmic vein and the right cavernous sinus. Thereafter, six flow diverters were deployed in the cavernous segment of the right ICA, with a significant reduction of the arteriovenous shunt (**b**). Early follow-up digital subtraction angiography (DSA) 6 d later revealed a residual arteriovenous shunt, which was treated with four additional flow diverters (**c**). Further DSA examinations 2 months (**d**) and 8 months (**e**) after the treatment showed the occlusion of the traumatic arteriovenous fistula (modified Rankin Scale 0)

FD placement was technically successful in all patients. There was no adverse event related to the FD placement in any of the patients. A total of 21 endovascular sessions were performed in 14 patients, with a single session in 8 patients, 2 sessions in 5, and 3 sessions in 1 patient (Fig. 1). Five patients were treated with FD alone at the first session and 2 patients with FD and covered stents. If coils were used in combination with FD in the same session, a transarterial approach was used for coil insertion and the FD was deployed thereafter (5 patients; Fig. 2). A primary transvenous approach for coil occlusion was used in 1 patient with a large shunt volume. In summary, a total of 59 FD (24 × Pipeline, Medtronic; 35 × p64, phenox), 291 bioactive detachable coils, and three stent grafts (Graftmaster, Abbott Vascular) were used in 21 procedures.

Of the 14 patients who underwent endovascular treatment, 3 exhibited a complete occlusion of the fistula after the initial treatment (21 %). A minor leak into the cavernous sinus was observed in 7 patients (50 %). In 4 patients, the shunt into the cavernous sinus was reduced significantly (29 %). No carotid occlusion, as salvage therapy, was necessary in the 14 cases. No procedural complications were encountered.

### Follow-up angiographic and clinical results

The median follow-up period encompassed 20 months (range 5–51 months). A total of 6/14 fistulae showed complete occlusion in the first angiographic control examination. One patient initially treated with FD alone needed a second embolization session consisting of transvenous coil occlusion of the cavernous sinus due to incomplete occlusion of the fistula. Access was gained via the inferior petrosal sinus. Two patients who were treated with FD alone in the initial treatment session also underwent a second session with additional FD placement, which led to a complete occlusion of both fistulae. Another 2 patients initially treated with FD and transarterial coil occlusion of the cavernous sinus also needed a second session with additional FD placement (*n* = 1) or flow diverter placement combined with further transvenous coil occlusion (*n* = 1). One patient with a high-volume fistula, initially treated with FD alone, needed two further treatment sessions with FD placement (second session) and transvenous coil insertion (third session) due to a persistent arteriovenous shunt.

Two patients experienced a thrombotic occlusion of the target ICA. In patient #1, the attempted stent percutaneous transluminal angioplasty (PTA) of a cavernous right ICA stenosis resulted in a diCCF, which was treated in the same session using two Graftmaster (Abbott Vascular, USA) and four FD. Follow-up digital subtraction angiography 7 months later confirmed the occlusion of the diCCF and the patency of the ICA. The dual platelet inhibition was interrupted 15 months later by another physician. MRI performed at another institution 16 months after the treatment and 1 month after the change in medication revealed the asymptomatic occlusion of the right ICA. In patient #11, the diCCF occurred after a severe head trauma together with a major intracranial hemorrhage. The endovascular procedure was carried out uneventfully several weeks later. Early follow-up DSA prior to discharge and 18 days after the last treatment showed thrombotic occlusion of the ICA. Analysis of the circumstances disclosed a lack of platelet function inhibition, related to a failure of the patient to comply with the medication regimen.

Of the 14 patients, 10 exhibited complete resolution of their ocular symptoms (71 %). Two patients reported residual symptoms with mild exophthalmos. No patient experienced worsening of preexisting or new symptoms.

## Discussion

The results of this series of patients with diCCF, treated by FD placement with or without coil insertion, indicate that the above-described treatment regimen is both safe and efficacious. After a median follow-up interval of 20 months, all diCCF were occluded and no patient had worsened clinically. Two patients showed an asymptomatic ICA occlusion during the follow-up DSA due to insufficient dual platelet antiaggregation. Nevertheless, it is a cost-intensive, extraordinary treatment that is at the moment off-label if the Pipeline embolization device is used.

The treatment of diCCF was for many years based on the deployment of initially latex and later silicone detachable balloons. After the market withdrawal of detachable silicone balloons in 2004, latex balloons remain available (Goldvalve, Balt Extrusion). The use of balloons for diCCF occlusion, albeit well established, was far from ideal. The transit of the balloon from the ICA lumen to the cavernous sinus can be difficult if the hole in the vessel wall is small. The inflation level of the balloon has to be well calibrated in order to occlude the fistula without compromising the patency of the ICA. Loss of the balloon from the microcatheter, displacement of the balloon during detachment, vessel dissection, cranial nerve compression in the wall of the cavernous sinus, and early balloon deflation are well-known issues [4, 13, 18, 23, 24]. Hence, these issues stimulated the development of numerous alternative endovascular treatment options for diCCF. Transarterial or transvenous coil occlusion, with or without balloon protection of the ICA, became popular. Dense packing of the cavernous sinus is key for this method. A long-term outcome study from Bink et al. in 2010 showed durable closure of CCFs and reliable regression of acute symptoms after coil embolization of 19 fistulae (13 direct and 9 dural CCFs). Nevertheless, 44 % of the patients had persistent cranial nerve deficits with disturbances of oculomotor and visual functions. The study group ascribed these persisting deficits to the underlying fistula size itself and/or the space-occupying effect of the coils, as there was a statistically significant correlation observed between coil volume and persistent diplopia and cranial nerve paresis [12]. To reduce the space-occupying effect of the coils, targeted occlusion of the fistulous compartment of the cavernous sinus became an option. Due to the complex anatomy of the cavernous sinus, this technique harbors the risk of incomplete fistula occlusion and thereby aggravation of the venous pressure within the orbital or cortical veins. Moreover, periprocedural protrusion of coil loops in the parent ICA could lead to thromboembolic events or even inadvertent occlusion of the ICA [25, 26]. As another treatment option, covered stents (sive “stent graft”) could be used. The Jomed Covered Stent Graft (Jomed International AB, Sweden, [27]), the Graftmaster Coronary Stent Graft System (Abbott Vascular) and the latest generation of pericardium-coated stent grafts (e. g., AneuGraft, ITGI Medical, Israel, [28]) are basically balloon-expandable stents with a covering membrane. Only AneuGraftNx has a CE mark for neurovascular indications. Covered stents have recently shown promising results in the treatment of diCCF [15, 29–31]. The key issues with balloon expandable stent grafts are the limited flexibility and the relatively high inflation pressure for the balloon, which may add trauma to the already injured ICA.

The use of liquid embolic agents such as Histoacryl (B. Braun, Germany), Onyx (Medtronic), or others for the treatment of diCCF involves the injection of these agents into the cavernous sinus. Uncontrolled propagation of the embolic agent, the unintended occlusion of efferent veins, and injury to the cranial nerves in the wall of the cavernous sinus are reported complications [18, 32, 33].

Not least, ICA occlusion with a balloon or with coils, or maybe with the newly available UNO Neurovascular Embolization System (Medtronic) [34], remains as a second-choice option if the anterior or posterior communicating artery collaterals provide sufficient cross flow to the dependent hemisphere.

FD are an extraordinary treatment option for diCCF, bearing in mind that their usage in the context of diCCF is off-label for Pipeline (Medtronic), Surpass (Stryker), and Fred (Microvention). The p64 (phenox) can be used on-label. In contrast to stent grafts, FD are significantly more flexible and are therefore easier to implant. They simultaneously provide a dense coverage of the hole in the arterial wall. Vessels originating from the covered part of the ICA are still supplied with blood due to the non-sealing but flow-diverting character of the FDs. Moreover, the vessel wall is redefined by the FD, thereby supporting the development of neointima. On the other hand, several disadvantages of this method are obvious. Only 5/14 diCCF in our series were cured with FD treatment alone. Especially in the case of a low-flow fistula, FD placement alone can lead to complete occlusion, whereas high-flow diCCF always need additional coil occlusion of the cavernous sinus to be cured. Moreover, this treatment method is cost intensive and there is often latency between FD treatment and occlusion of the diCCF. Additionally, the necessity of stringent dual platelet inhibition for at least several months, if not forever, is another drawback of this treatment method.

A dedicated self-expanding stent graft might have the advantage that a single implant could be sufficient, thus reducing the costs of treatment and allowing for an instantaneous occlusion of the diCCF.

## Conclusion

FD deployment is a technical option for the treatment of diCCF. The procedure per se is straightforward. A combination with coil occlusion of the cavernous sinus is possible. The deployment of several FD and possibly several treatment sessions may be necessary. Complete diCCF occlusion may take several weeks. Injury to the cranial nerves in the cavernous sinus is avoided. Most patients will improve clinically, but strict adherence to dual platelet function inhibition is key. A dedicated self-expanding stent graft is the awaited solution for several of the above-mentioned issues.

## Abbreviations

CT: computed tomography
diCCF: direct carotid-cavernous sinus fistula
DSA: digital subtraction angiography
FD: flow diverter
ICA: internal carotid artery
ID: inner diameter
IV: intravenous MRA magnetic resonance angiography
PO: per os* PTA* percutaneous transluminal angioplasty
